# Supplementary material for: Brain MRI findings in relation to clinical characteristics and outcome of tuberculous meningitis
Source: PLoS One. 2020 Nov 13;15(11):e0241974. doi: 10.1371/journal.pone.0241974 (PMC7665695; doi:10.1371/journal.pone.0241974)
Supplement: Attachment — Submitted filename: Response to the Reviewers_Appeal.docx [file pone.0241974.s002.docx]

**Authors’ Response to the Review Comments**

| Journal | Plos One |
| --- | --- |
| Manuscript# | PONE-D-19-33449R1 |
| Title of Paper | Brain MRI findings in relation to clinical characteristics and outcome of tuberculous meningitis |
| Authors | Sofiati Dian, Robby Hermawan, Arjan van Laarhoven, Sofia Immaculata, Tri Hanggono Achmad, Rovina Ruslami, Farhan Anwary, Ristaniah D. Soetikno, Ahmad Rizal Ganiem, Reinout van Crevel |

**Response to Comments from Reviewer #1**

**Comments:**

**All concerns were addressed by the authors. The response to reviewers’ concerns is point by point argue and clear. I am agree with their decision to not improved the references.**

**For me the manuscript is suitable for publication.**

We would like to thank Dr. Bleibtreu for the positive appraisal of our manuscript.

**Response to Comments from Reviewer #2**
**Comments:**

**In this study, the authors used MRI data collected at baseline in 48 patients with tuberculous meningitis (TBM). They then attempted to test the association between MRI findings (which included meningeal enhancement, tuberculomas, brain infarctions, and hydrocephalus).and clinical characteristics (motor deficits, cranial nerve palsy, microbiological confirmation, and CSF abnormalities). This analysis, however is flawed because none of these comparisons are “clean.” This is due to the fact that so many of the patients had multiple MRI abnormalities. It doesn’t make sense to test each abnormality separately against the others and make conclusions about an individual abnormality given that there are a large number of possible permutations of abnormalities that could occur in any given patient. There is no way to be certain that any one abnormality is associated with any clinical characteristic, since the abnormalities so frequently occur in combination.**

Thanks for including a separate statistical review, bringing up relevant point. Indeed many of the associations in these clinical studies are ‘unclean’, as pointed out, i.e. in previous report by Thwaites et.al, Kalita et.al, and Wasay et.al, many events occur together. Moreover, due to its relative infrequent nature, series of tuberculous meningitis patients are often small. Among all typical brain MRI findings, none has consistently been linked with a bad outcome. Therefore, to make it sure, we weighted the abnormalities based on the total number of it: ‘no abnormalities’, a single abnormality, or 2, 3 or 4 abnormalities (table 2), showing that the presence of 4 abnormalities is associated with more severe clinical presentation, bacteriological confirmation and CSF inflammation.

**The way to do this correctly would be to create a model in which the presence or absence of the clinical characteristic is the outcome, and a binary variable is created for each of the potential MRI abnormalities (allowing for one subject to have multiple abnormalities). It is doubtful that there are enough data to give sufficient power to such an analysis. Furthermore, it would also be advisable to consider interactions between the MRI findings (e.g., do patients with both meningeal enhancement AND tuberculomas have a different relationship with a clinical finding than patients who have meningeal enhancement WITHOUT tuberculomas). The small sample would clearly lack sufficient power to see any sort of interactions. I don’t think there is any way this can be fixed.**

We agree with the reviewer that sample size is a concern (line 286). Still, this study represents serial MRI analysis of the 2^nd^ largest cohort of well characterized and very high proportion of bacteriologically proven disease of TBM patients, a disease with a 1% incidence among human tuberculosis patients. In response to the reviewer’s comment, we now stress more clearly that the associative analysis of neuroradiology findings with the clinical and laboratory features is of an explorative nature (line 50, 89, 186, 227, 269). We also incorporated the suggestion of weighting the brain abnormality by the number of the finding (table 2).

**The same problem is inherent in the analysis of the effect of brain abnormalities on six-month mortality and functional outcome. It isn’t correct to examine each one separately, for the above reasons. A model would need to contain a variable (yes/no) for EACH abnormality. One possible option would be to count the number of brain abnormalities any patient had, and check whether the number of abnormalities was associated with the different clinical outcomes. I’m not sure this would yield useful information, however.**

Regarding this study more as descriptive and exploratory, a complete statistical model – also given the study size – might not be appropriate. However, following the reviewer’s suggestion we have examined a possible association between the number of MRI abnormalities, paradoxical response and patient outcome (table 2 and table 4). This shows a comparable number of patients who died or had a good recovery after 6-month of treatment among groups with different number of brain abnormalities. However, good recovery after 6-month of treatment seems more common in group of patients with only radiological or had no paradoxical response.

**It is not surprising that the authors found none of the MRI abnormalities (again, examined separately) were associated with mortality, disability, or worsening. There is too much overlap between any “one” abnormality and “all others,” since very few subjects HAD just one abnormality. Again, it requires a much more complex analysis than is possible given the small size of the data.

I feel that the only way these data could be presented would be descriptively, without an attempt to derive conclusions from very oversimplified (and incorrect) analyses**.

Following the reviewer’s suggestion, we now present the data in a more descriptive way. We have rephrased several statements to make it less strong in the abstract (lines 50), introduction (line 86), results (lines 186) and discussion (lines 227, 269):

**Minor points:
The authors state that 60 patients were enrolled in the “parent” study (a Phase 2b dose-finding study). That is true, but if 12 of these did not have a baseline MRI, then they would never have been considered for the current study. A better way to present this would be to say that of 60 patients enrolled in a Phase 2b dose-finding clinical trial, 48 had baseline MRIs and were therefore eligible for the current study.**

Thanks for this suggestion, we have rephrased this sentence (line 167-171).

**l.162 Multivariable, not multivariate**

Thank you for pointing out this mistake; we deleted this sentence altogether because we changed the analysis.

**Survival analysis, not logistic regression, should be used to look at six-month mortality, but again, the fact that subjects could have multiple MRI abnormalities would have to be accounted for.**

We thank the reviewer for pointing this out. Indeed, it will be less accurate to predict the mortality by cox or logistic model using this single brain lesion due to the ‘unclean’ variable of each brain abnormality as pointed out by the reviewer. As suggested and a previously applied (Kalita, et.al) we adopted to a descriptive approach (table 2). in a larger study, this approach might have been used (for instance as done by Wasay et.al(3) in their study), but we have now used a more descriptive approach as Kalita, et.al(2) have done (table 2).

We again thank the editor and reviewers for their time and efforts to help improve our manuscript which we hope is now suitable for publication.

**Reference**

1.	Thwaites GE, Macmullen-Price J, Tran TH, Pham PM, Nguyen TD, Simmons CP, et al. Serial MRI to determine the effect of dexamethasone on the cerebral pathology of tuberculous meningitis: an observational study. Lancet Neurol. 2007;6(3):230-6.

2.	Kalita J, Prasad S, Misra UK. Predictors of paradoxical tuberculoma in tuberculous meningitis. Int J Tuberc Lung Dis. 2014;18(4):486-91.

3.	Wasay M, Farooq S, Khowaja ZA, Bawa ZA, Ali SM, Awan S, et al. Cerebral infarction and tuberculoma in central nervous system tuberculosis: frequency and prognostic implications. J Neurol Neurosurg Psychiatry. 2014;85(11):1260-4.

4.	Anuradha HK, Garg RK, Agarwal A, Sinha MK, Verma R, Singh MK, et al. Predictors of stroke in patients of tuberculous meningitis and its effect on the outcome. QJM. 2010;103(9):671-8.

5.	Singh AK, Malhotra HS, Garg RK, Jain A, Kumar N, Kohli N, et al. Paradoxical reaction in tuberculous meningitis: presentation, predictors and impact on prognosis. BMC Infect Dis. 2016;16(1):306.
